# Nitric Oxide Concentration and Other Salivary Changes after Insertion of New Complete Dentures in Edentulous Subjects

**DOI:** 10.1155/2016/8351427

**Published:** 2016-02-29

**Authors:** Maria de Lourdes Breseghelo, Lídia Andreu Guillo, Túlio Eduardo Nogueira, Cláudio Rodrigues Leles

**Affiliations:** ^1^Department of Biochemistry and Molecular Biology, Biological Sciences Institute, Federal University of Goias, Avenida Universitária Esquina com 1a Avenida s/n, Setor Universitário, 74605-220 Goiania, GO, Brazil; ^2^Department of Oral Prevention and Rehabilitation, School of Dentistry, Federal University of Goias, Avenida Universitária Esquina com 1a Avenida s/n, Setor Universitário, 74605-220 Goiania, GO, Brazil

## Abstract

*Objective*. To assess changes in levels of salivary nitric oxide (NO) after insertion of new complete dentures and its association with clinical and salivary parameters.* Methods*. Nineteen fully edentulous subjects were included, mean age 64.4. Unstimulated whole saliva was collected before and after insertion of the dentures, at follow-up visits, and after 12 months. The concentration of the final stable NO product (nitrite) was measured by a colorimetric assay based on the Griess reaction. Clinical parameters were assessed during all clinical visits.* Results*. Functional adaptation to the dentures progressively improved, with no complaints at the long-term follow-up. NO concentration was not influenced by the level of functional adaptation, presence of injuries to the mucosa, salivary flow, and saliva viscosity. Pairwise comparison showed a reduction in NO concentration at the first follow-up compared to baseline values but differences were not statistically significant. Significant differences were observed in NO concentrations at the long-term follow-up when compared to the first (*p* = 0.024) and second (*p* = 0.027) visits.* Conclusion*. NO concentration reduced after denture insertion and returned to baseline levels in the long-term follow-up. This appears to be an autonomic response of the body and provides valuable complementary information for the management of the edentulous patient.

## 1. Introduction

Conventional complete denture is the most common treatment for edentulous patients and most of them are well adapted to their dentures with no significant complaints [1]. However, some transitory problems may arise after the insertion of new dentures, which may last for one or two weeks, causing different levels of pain, discomfort, and functional difficulties. Even when the dentures achieve all technical and clinical prosthodontic requirements, some patients are unable to adapt to them, and this initial period can be a relevant source of dissatisfaction with treatment, when not properly managed by the patient and prosthodontist [2].

Factors that influence patient's adaptation to the dentures are not fully understood. Previous studies explored factors such as quality of dentures, oral condition, patient-dentist relationship, attitudes toward dentures, patient's personality, and socioeconomic factors. However, few of these factors are only weakly correlated with patient satisfaction with the dentures, which is individually determined and is often unpredictable for both the patient and prosthodontist [3].

In older edentulous subjects, low salivary secretion due to advanced age, use of medicaments, and systemic physiological degeneration may predispose individuals to oral mucosa diseases, and such age-related changes must be considered during prosthodontic treatment [4]. When saliva flows in adequate amount and consistency, it favors physical mechanisms that contribute to a satisfactory retention of the dentures [5]. Moreover, saliva forms a layer inside the mouth that promotes the protection of oral tissues, permits its hydration, and is also considered the body's first line of natural defense [6, 7].

The use of prosthesis may also be a predisposing factor for the onset of salivary changes that affect oral homeostasis and oral mucosal health and have detrimental influence on oral health-related quality of life, especially in older subjects who are the most prevalent group of edentulous patients and denture wearers [8]. During the first days after insertion of new dentures, sore spots or injured mucosa are commonly observed. Some studies report changes in saliva composition during this period, particularly the concentration of acid uric and pH levels [8, 9]. However, it is still unclear if these changes have an impact on the patient's adaptation to the denture and which salivary components play a relevant role in this process.

Among saliva constituents, the nitric oxide (NO) is a biochemical marker associated with the extent of inflammatory diseases such as gingivitis and periodontitis [10] and, predictively, the treatment of periodontal disease decreases NO production levels [11]. NO is a signaling molecule synthesized by a myriad of cell types and it is responsible for regulating immune function, blood vessel dilatation, and neurotransmission [12]. It is produced by the enzyme nitric oxide synthase (NOS) [13]. High concentrations of nitrate and nitrite (stable metabolites of NO) in normal saliva may ensure potentially protective effects, such as antibacterial properties, increased mucosal blood flow, and oral mucus production [14]. Recent findings have confirmed their involvement in physiological and pathological processes of the salivary glands [15, 16], and studies in animal models have demonstrated the mediation of nitric oxide in protein secretion by acinar cells after parasympathetic and sympathetic stimulation [17, 18].

In order to explore other clinical variables that would influence patient response to complete denture treatment, we hypothesized that difference in clinical adaptation to the new dentures may be moderated by biochemical factors correlated with inflammatory process and stress. Hence, the aims of this study were to measure changes in salivary NO levels before and after insertion of new complete dentures and to verify its association with clinical and salivary parameters (pH, flow, and viscosity).

## 2. Material and Methods

The study sample included a consecutive group of fully edentulous subjects needing replacement of complete maxillary and mandibular dentures. Participants should present good general health and no local or medical contraindications for treatment. In addition, individuals were expected to be mentally and physically healthy. The institutional ethical committee previously approved the study protocol and an informed consent was obtained from each participant.

For participants included in the study, new conventional complete dentures were fabricated following the traditional protocol for denture construction. Unstimulated whole saliva (UWS) samples were collected before denture insertion, during subsequent appointments for adjustments, and after a 10- to 12-month period. To perform saliva collection, before the prosthodontic appointment, a sterilized cylindrical cotton piece was placed in the sublingual region for 4 minutes, and then it was removed and compressed with a disposable 5 mL syringe into a 2.0 mL sterile microcentrifuge tube. When the required 1.5 mL volume was not reached, the procedure was repeated. Samples were centrifuged at 8000 rpm for 4 min at 4°C, after which 0.5 mL aliquots of the supernatant was transferred to new, sterile microcentrifuge tubes. The tubes containing saliva were identified with the patient's initials, date of collection, and treatment stage. Subsequently, they were stored at −20°C to avoid bacterial growth.

Salivary NO concentration was determined by quantifying the stable end product of NO, nitrite. A colorimetric assay kit (Molecular Probes, Eugene, OR, USA) based on Griess reaction [20] was used according to the manufacturer's instructions. A previous pilot study indicated that saliva samples could be used without any predilution. Briefly, 150 *μ*L aliquots was mixed in triplicate with 20 *μ*L of freshly prepared Griess reagent [one volume of 1% (w/v) sulfanilamide in 5% phosphoric acid and one volume of 0.1% (w/v) N-(1-naphthyl) ethylenediamine dihydrochloride] and with 130 *μ*L of distilled water in a microplate. The mixture was kept at room temperature for 30 minutes. The absorbance of the purple chromophore was measured at 540 nm in a microplate reader (ELx800, Bio-Tek Instruments, Highland Park, VT, USA). Nitrite concentrations were calculated from a standard curve constructed using the sodium nitrite stock solution provided by the kit. Additionally, salivary pH was measured using a pH strip and recorded by comparing the strip to a pH scale.

The following clinical parameters were assessed and classified in all clinical visits: level of functional adaptation with the dentures, presence of injuries in the mucosa, salivary flow, and salivary viscosity. Classification criteria for these variables are described in Table 1.

Data were presented in terms of median, range, and mean (and standard error, SE). Data analysis included changes in the clinical and salivary parameters before and after insertion of new dentures at the follow-up appointments. The nonparametric Wilcoxon matched-pairs signed rank test and the Kruskal Wallis test were used for group comparisons at the 0.05 level of significance. IBM-SPSS 20.0 (Chicago, IL, USA) and GraphPad Prism 5.0 (San Diego, CA, USA) were used for data analysis.

## 3. Results

**Figure 1:**
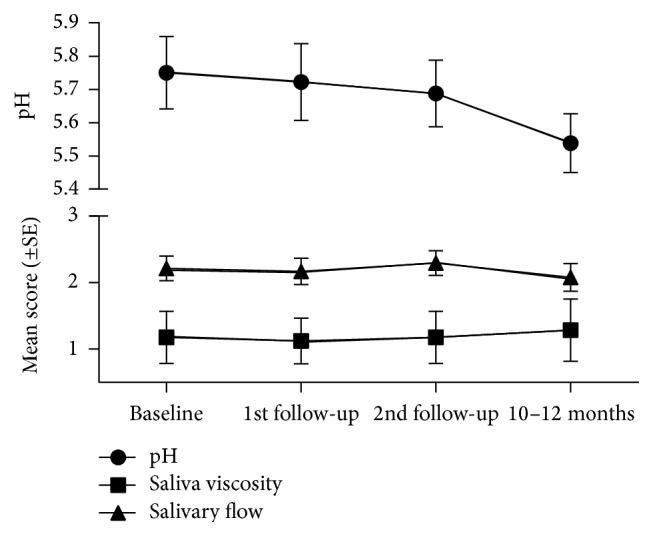
Changes in salivary viscosity, visual flow, and pH measures before and after insertion of new dentures at follow-up appointments. Values are represented as mean and standard error (SE).

Nineteen edentulous subjects were included in this study: 14 females and 5 males, mean age 64.4 years (SD = 8.3). Measures of salivary visual flow, viscosity, and pH of the unstimulated whole saliva were evaluated during the adaptation period and data are summarized in Figure 1. At the last follow-up (10–12 months after insertion) pH values decreased, although the differences between insertion (5.75 ± 0.11) and the last follow-up (5.54 ± 0.09) were not significant (*p* = 0.172). Changes in viscosity and salivary flow were also not statistically significant.

**Figure 2:**
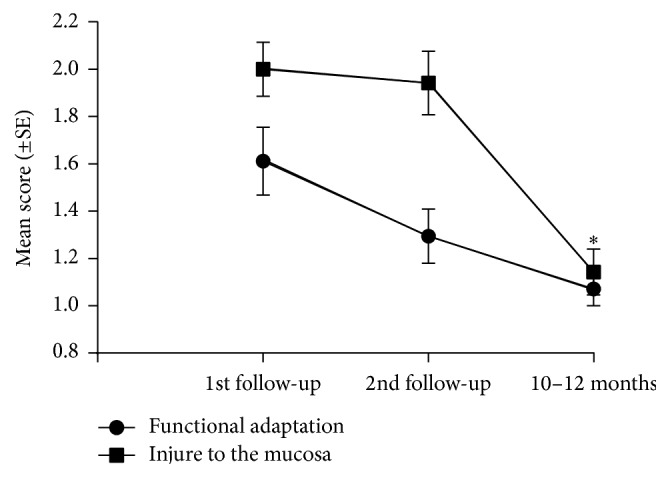
Levels of functional adaptation and scores of injuries to the mucosa assessed in the follow-up appointments (*n* = 19). Values are presented as mean and standard error (^*∗*^ *p* < 0.05; Wilcoxon matched-pairs signed rank test).

As shown in Figure 2, difficulties related to the functional adaptation and the number of injured areas in the mucosa decreased after the initial follow-up visit. The mean score of functional adaptation at the first follow-up visit (1.61 ± 0.14) was significantly higher than at the end of the observation period (1.07 ± 0.07) (*p* = 0.015). Moreover, the scores of injuries to the supporting mucosa were significantly lower at the end of the evaluation period (*p* = 0.001).

**Figure 3:**
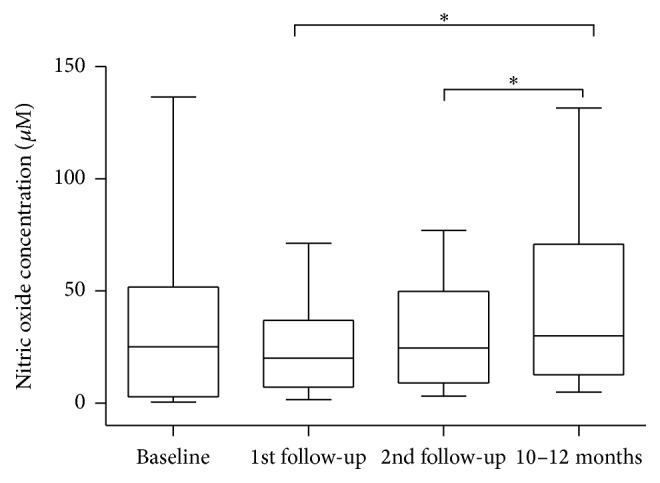
Box-whisker plot of changes in nitric oxide levels (*μ*M) in unstimulated whole saliva during follow-up sessions after insertion of new complete dentures (*n* = 19). Box limits represent 25 and 75 percentiles, the line within the box represents median, and the whiskers represent minimum and maximum values (^*∗*^ *p* < 0.05; Wilcoxon matched-pairs signed rank test).

Changes in NO concentration are shown in Figure 3. Mean salivary NO levels at the insertion session were higher than at the first and second visits, although differences were not statistically significant (39.51 ± 11.79 *μ*M versus 25.89 ± 5.54 *μ*M; *p* = 0.455 and 39.51 ± 11.79 *μ*Mversus 30.42 ± 6.76 *μ*M; *p* = 0.6355, resp.). NO levels were significantly higher at the 10 to 12-month follow-up compared to the first (*p* = 0.024) and second (*p* = 0.027) follow-up visits. No difference was observed between the mean NO levels at baseline and at the last follow-up (39.51 ± 11.79 *μ*M versus 44.81 ± 10.78 *μ*M; *p* = 0.423).

The samples from patients with injuries to the mucosa scored as 1, 2, and 3 showed mean NO levels of 41.18 ± 9.63 *μ*M, 28.31 ± 4.72 *μ*M, and 27.22 ± 10.14 *μ*M, respectively, during the period of treatment. We observed that the differences in mean NO level in relation to injuries to the mucosa were not statistically significant (*p* = 0.673), but higher NO levels were observed in samples with no injuries (score 1).

No differences in NO levels were observed when the different levels of functional adaptation to the dentures were compared (*p* = 0.391). Similarly, NO levels were not influenced by the salivary viscosity (*p* = 0.289) and by the salivary visual flow (*p* = 0.729).

## 4. Discussion

Although the main sources of clinical problems in maladaptive patients are easily identifiable in clinical practice, some physiological mechanisms of the changes that occur during this period are not completely known. This study investigated changes in the saliva composition of edentulous patients after insertion of new complete dentures. Nineteen individuals were included and during the evaluation period no statistically significant differences were found in relation to salivary physical parameters, including pH, viscosity, and visual flow.

Previous investigations had conflicting conclusions with respect to changes in saliva composition after the insertion of new dentures. Bhat et al. [7] found decreased pH salivary levels, whereas Nikolopoulou and Tzortzopoulou [9] observed higher pH values 15 days after the insertion of either conventional or implant-supported dentures. In our study, there were no significant differences of pH throughout the period under evaluation (*p* = 0.172) and this finding is in agreement with Mäkilä [21]. It is important to mention that distinct pH assessment methods were used among studies (pH strips versus an electronic device) and this could be a reason for the different conclusions.

In our study, salivary flow rating remained constant with no significant differences over time. Unlike other investigations, we evaluated it through a visual inspection test, which consisted of observing, under satisfactory illumination, the time required for formation of saliva droplets over the lower lip mucosa. The use of this specific method made it difficult to compare these findings with other studies, since only the time for saliva formation was evaluated, not the amount of saliva.

We also observed that the level of adaptation difficulties and the amount of trauma significantly decreased, possibly indicating that subjects were able to adapt to the new denture (Figure 2). Initial difficulties gradually diminished over time, 68.4% of patients had a favorable outcome at the short-term follow-up, and almost all patients (95%) reported no complaints and a favorable adaptation at the long-term follow-up.

All patients included in our study were experienced denture wearers, so we would expect that the replacement of dentures would be well accommodated. The functional adaptation process begins immediately after insertion of the dentures and the transitory difficulties that usually arise are related to initial foreign body sensations in the mouth, phonetic changes, excessive salivation, difficulty chewing, and coordinating the jaw, as well as possible trauma associated with improper extension of denture borders or localized areas of overcompression [22, 23]. A comprehensive approach that takes into account not only technical aspects of the denture construction but also motivation, communication, and empathy in the dentist-patient relationship are essential parts of a successful treatment [2, 24, 25].

Changes in the salivary NO concentrations were statistically and significantly different when comparing levels of the final evaluation (higher concentration) to the first and second adjustment visits. In general, NO concentration decreased during the adaptation period and after 10–12 months; it returned to levels close to baseline. This finding might indicate an autonomic body response that culminated in lesser stimulation (or inhibition) of nitric oxide synthase activity. We hypothesized that this effect could be verified because patients were edentulous and the interference of teeth in NO levels could be eliminated. In addition, the fact that no relevant infectious process or large traumatic lesions were observed during the short-term follow-up period may be the reason why the nitric oxide synthase enzyme was not activated in oral cavity cells and salivary glands.

Nitric oxide plays an important role in the destruction of periodontal tissue in periodontitis [11]. The association between NO and the etiopathogenesis of periodontitis has been reported [11] as well as the potential capacity of NO to orally defend against caries pathogens [26]. Studies in patients with chronic periodontal disease showed that nitric oxide is important to host defense and homeostasis, and it modulates the inflammatory response in periodontitis, leading to detrimental effects on invading microbes. It plays an important and complex role in inflammatory immune processes and in the remodeling and maintenance of bone structures [27]. Hence, salivary nitrite increases in patients with periodontal disease and may be associated with such defense mechanisms. However, there is no consensus to date, since decreased levels of NO have also been reported [28].

Studies involving nitric oxide analysis in edentulous patients are very scarce. To our knowledge, only one study reported that salivary nitric oxide concentrations are significantly higher in healthy, although fully edentulous, patients in relation to partially edentulous patients with implant-supported overdentures [29]. These authors suggested that the accuracy of the study results were improved in fully edentulous patients because teeth may influence the results as previously proposed by Liskmann et al. [30]. Rocha et al. [29] observed that healthy edentulous patients present higher levels of nitric oxide than patients with peri-implant inflammation, which corroborated our results. Further research is still needed to confirm our results, since a limitation of this study was the small number of patients.

In summary, salivary NO concentrations (in its oxidized form as nitrite) change in response to an adaptive period after insertion of new complete dentures. A reduction in NO concentration levels was observed immediately after denture insertion, which returned to baseline levels in the long-term follow-up. The decreased NO levels appear to be an autonomic response of the body to the decreased salivary flux and mucosa ulceration, two important clinical parameters that can be easily monitored. So, an improvement in treatment outcome could be obtained if nitric oxide levels were assessed in parallel with the clinical evaluation of injuries to mucosa.

## Figures and Tables

**Table 1:** Classification of clinical parameters.

Variable	Categories	Criteria
Level of functional adaptation to the dentures^**∗**^	1	Report of favorable adaptation and continuous use of the dentures; absence of or minimal complaints
	2	Report of difficult adaptation or intermittent use of the dentures; moderate to severe complaints
	3	Report of extreme difficulties and sporadic or no use of the dentures; severe complaints

Injuries to the supporting mucosa^**∗**^	1	Absent
	2	1 or 2 sore spots or ulceration evidenced by visual inspection, digital pressure or tissue compression with the denture
	3	Multiple sore spots or ulcerations evidenced by visual inspection, digital pressure, or tissue compression with the denture

Salivary flow rating^*∗∗*^	Low = 1	Greater than 60 seconds
	Normal = 2	Between 30 and 60 seconds
	High = 3	Less than 30 seconds

Saliva viscosity^§^	1	Watery and transparent
	2	Thick and sticky
	3	Sparkling

^*∗*^Developed by the authors.

^*∗∗*^Visual inspection test, which consisted of observing, under satisfactory illumination, and the time required for formation of saliva droplets over the lower lip mucosa [19].

^§^Based on the criteria proposed by the Saliva-Check Buffer Testing Mat. GC America, 2016.
